# Characterization of Plasmids in a Human Clinical Strain of *Lactococcus garvieae*

**DOI:** 10.1371/journal.pone.0040119

**Published:** 2012-06-29

**Authors:** Mónica Aguado-Urda, Alicia Gibello, M. Mar Blanco, Guillermo H. López-Campos, M. Teresa Cutuli, José F. Fernández-Garayzábal

**Affiliations:** 1 Faculty of Veterinary Sciences, Department of Animal Health, Complutense University, Madrid, Spain; 2 Bioinformatics and Public Health Department, Health Institute Carlos III, Madrid, Spain; 3 Animal Health Surveillance Center (VISAVET), Complutense University of Madrid, Spain; Belgian Nuclear Research Centre SCK/CEN, Belgium

## Abstract

The present work describes the molecular characterization of five circular plasmids found in the human clinical strain *Lactococcus garvieae* 21881. The plasmids were designated pGL1-pGL5, with molecular sizes of 4,536 bp, 4,572 bp, 12,948 bp, 14,006 bp and 68,798 bp, respectively. Based on detailed sequence analysis, some of these plasmids appear to be mosaics composed of DNA obtained by modular exchange between different species of lactic acid bacteria. Based on sequence data and the derived presence of certain genes and proteins, the plasmid pGL2 appears to replicate via a rolling-circle mechanism, while the other four plasmids appear to belong to the group of lactococcal theta-type replicons. The plasmids pGL1, pGL2 and pGL5 encode putative proteins related with bacteriocin synthesis and bacteriocin secretion and immunity. The plasmid pGL5 harbors genes (*txn, orf5* and *orf25*) encoding proteins that could be considered putative virulence factors. The gene *txn* encodes a protein with an enzymatic domain corresponding to the family actin-ADP-ribosyltransferases toxins, which are known to play a key role in pathogenesis of a variety of bacterial pathogens. The genes *orf5* and *orf25* encode two putative surface proteins containing the cell wall-sorting motif LPXTG, with mucin-binding and collagen-binding protein domains, respectively. These proteins could be involved in the adherence of *L. garvieae* to mucus from the intestine, facilitating further interaction with intestinal epithelial cells and to collagenous tissues such as the collagen-rich heart valves. To our knowledge, this is the first report on the characterization of plasmids in a human clinical strain of this pathogen.

## Introduction

*Lactococcus garvieae* is a ubiquitous and widely distributed microorganism that has relevance in veterinary and human medicine. The increasing number of cases of human infections by *L. garvieae* reported in recent years have caused it to be considered an opportunistic emerging human pathogen. The most common manifestation is infective endocarditis involving either native or prosthetic valves [1]–[4], but it has also been associated with different clinical processes such as septicaemia, urinary infections and skin infections in healthy and immunocompromised patients [5]–[7]. *L. garvieae* is also an important bacterial fish pathogen responsible for lactococcosis, a septicemic infection affecting various wild and farmed fish species, particularly in trout [8]. It has also been isolated from clinical specimens in other animal species, such as cows and water buffalos with subclinical mastitis and pigs with pneumonia, and from cat and dog tonsils [8]–[10]. *L. garvieae* has also been found in vegetables, meat and sausages, but mainly in artisanal dairy products [11]–[13]. Because of this, *L. garvieae* is considered a potential emerging zoonotic pathogen [14], [15].

Recently, the complete genome sequences of four clinical strains of *L. garvieae* isolated from yellowtail and trout [16]–[19], one human clinical strain [20] and one dairy strain [19] have been published. A preliminary genome comparison of the fish and human strains of *L. garvieae* 8831 and 22881, respectively, showed a difference in their genome sizes of approximately 0.1 Mb [16], [20]. As shown in the present work, this difference is related to the presence of five plasmids found in the human strain. Plasmids are commonly found in many members of lactic acid bacteria [21]–[24], encoding relevant properties such as additional amino acid and carbohydrate metabolism, proteolysis activities, exopolysaccharide biosynthesis, bacteriophage resistance, bacteriocin production, drug resistance or virulence factors. Unlike other species of *Lactococcus*, such as *Lactococcus lactis* and *Lactococcus salivarius*, in which the genetic content of different plasmids have been studied and characterized [24], [25], the data for *L. garvieae* are very limited. Fortina *et al*. [26] observed that *L. garvieae* strains of dairy origin often harbor plasmids, and Reimundo *et al*. [27] reported the presence of a 30-kb plasmid in a human clinical strain; however, neither study characterized the plasmids. There is only one study that characterized a conjugative plasmid carrying multiple drug resistance genes in *L. garvieae* strains that were isolated from yellowtails [28]. In this work, we present the sequence analysis and characterization of five circular plasmids found in the human *L. garvieae* strain 21881.

## Materials and Methods

### Bacterial Strains and Growth Conditions

*Lactococcus garvieae* 21881 was isolated from the blood of a 74-year-old male patient affected by septicaemia [20]. The bacteria were grown in MRS broth (Cultimed, Panreac Laboratories) and incubated at 30°C for 24 h. The strain 8831, isolated in Spain in 2004 from diseased trout affected by lactococcosis [16], was used as a reference.

### Plasmid Isolation and Plasmid Stability Tests

The isolation of plasmid DNA was performed using log-phase cultures (OD_600_, ∼1) using the method of Anderson and McKay [29]. The plasmid profile was observed by electrophoresis of 15 µL of each sample on a 0.6% (w/v) agarose gel supplemented with 1X Syber safe® (Invitrogen, Eugene, OR, USA).

The stability of the plasmids was determined after growing the cells in MRS liquid cultures for approximately 100 generations. Briefly, one colony of *L. garvieae* 21881 was grown overnight in MRS broth for 16 h (approximately 20 generations) for a total of 5 days. After this time without selection (approximately 100 generations), 50 colonies were picked and patched onto MRS agar plates (MRS broth supplemented with 1.5% agar). The patches were tested for plasmid isolation, and the plasmid stability was calculated as the percentage of patches or clones in the population that maintained the plasmid content.

### DNA Manipulation

PCR primers for gap closure were designed based on DNA sequence information at the end of the corresponding contigs. In vitro amplification reactions of DNA were performed in a reaction mixture of 100 µL containing DNA template (10–20 ng), 1 µM of each primer, 100 µM of each dNTP (Biotools), 5 U of Ultratools DNA polymerase (Biotools) and its 1X amplification buffer. The amplifications were performed in a Mastercycler gradient thermal cycler (Eppendorf) following the optimal cycle profile: an initial denaturation step of 95°C for 5 min, 35 serial cycles of a denaturation step of 95°C for 45 seconds, annealing at the optimal annealing temperature corresponding to each primer set for 1 min and extension at 72°C for 2 min, followed by a final extension step of 72°C for 10 min. Negative controls (no DNA template) were included in each batch of PCR reactions. PCR-generated products were detected by electrophoresis of 5 µL of each amplification mixture on 1% agarose gels supplemented with 1X Syber safe®. The amplicons were purified (Geneclean Turbo Kit, MP Biomedicals LLC) and sequenced at SECUGEN facilities (Centro de Investigaciones Biológicas, CSIC, Spain) using the DyeDeoxy (dRhodamine) Terminator Cycle Sequencing kit in an automatic ABI Prism DNA sequencer.

### Plasmid DNA Sequencing, Sequence Assembly and Annotation

The sequences of the five plasmids of *L. garvieae* 21881 were obtained from published whole genome sequencing data [20]. The whole sequences corresponding to each plasmid were completed by PCR reactions, which allowed us to join and fill the gaps of contigs: c42, c41 and c80 (pGL5); c20 (pGL4); c53 (pGL3); c102 (pGL2) and c101 (pGL1).

Open reading frames (Orfs) were identified using a combination of the GeneMark.hmm for prokaryotes (v2.8) program [30] with the *Lactococcus lactis* genome as a reference (http://exon.gatech.edu/gmhmm2_prok.cgi), and the ORF Finder tool (http://www.ncbi.nlm.nih.gov/projects/gorf/) from the National Center for Biotechnology Information (NCBI). Potential Orfs were subsequently manually filtered using the following criteria: i) only Orfs whose DNA sequences did not overlap with those of other Orfs or doing so in less than 21 nucleotides were considered, and ii) only Orfs larger than 50 amino acids (aa) were considered. The amino acid sequences of selected proteins were further analyzed using the BLASTp program and the NCBI’s nonredundant protein database. Putative functions were assigned on the basis of the best BLASTp hit on an annotated protein. The predicted proteins were functionally categorized by using the clusters of orthologous groups (COG), conserved domain (CDD), the TIGR Gene Indices and the protein family (pfam) databases.

Figures of physical and genetic maps of plasmids pGL1-pGL5 and DNA sequence similarities with plasmids of other Gram-positive bacteria were generated by means of the BLAST Ring Image Generator (BRIG) using the default parameters [31].

Signal peptides and cleavage sites in Gram-positive bacteria amino acid sequences were predicted using the SignalP 4.0 Server [32] available at http://www.cbs.dtu.dk/services/SignalP/.

### Horizontal Gene Transfer (HGT) Analysis

To identify putative horizontally transferred genes, sequence composition analysis was performed for some genes, including the calculation of GC composition and dinucleotide dissimilarity value (δ*) using the δρ-WEB tool (http://deltarho.amc.uva.nl/cgi-bin/bin/start.cgi). A high genomic dissimilarity between an input sequence and a representative genome sequence of the species from which the sequence was isolated suggests a heterologous origin of the input sequence and this difference can be expressed by the δ* value [33]. DNA fragments with different GC composition and/or a high dissimilarity value compared with those of the whole genome of *L. garvieae* 21881 were predicted to be HGT genes. The predicted horizontally transferred genes and gene clusters were checked for HGT mechanism-associated features such as neighboring mobile elements. Homologous sequences were aligned with MUSCLE [34], and phylogenetic trees were constructed using Phylogeny.fr [35]. The positions of orthologs from *L. garvieae* and other acid lactic bacteria in the phylogenetic trees were checked to confirm whether the predicted genes are horizontally transferred between genomes.

### Susceptibility to Quinolones

The antimicrobial susceptibility to nalidixic acid, oxolinic acid, flumequine, ciprofloxacin, norfloxacin and moxifloxacin of the strain *L. garvieae* 21881 was determined by the disk diffusion method using commercially prepared antimicrobial disks (Oxoid, Ltd.). Inoculum was prepared from a 48 h Columbia blood agar plate by suspending four colonies in 5 mL of PBS and adjusted to a 0.5 McFarland standard. The disk diffusion test was performed as described by the Clinical and Laboratory Standards Institute [36]. The Mueller-Hinton-blood agar plates were examined after 24 h of incubation at 30°C. *Staphylococcus aureus* ATCC 25923 and *Escherichia coli* ATCC 25922 were included as quality control. As no specific inhibition zone diameter (IZD) breakpoints for *L.garvieae* are available, the IZD breakpoints were those recommended by the French Society of Microbiology [37] for testing Gram-positive microorganisms.

The amino acid sequences of GyrA, GyrB, ParC and ParE were obtained from their respective genes located in contigs 61, 39, and 61 [20]. The amino acid sequences of GyrA and ParC from *L. garvieae* 21881, including the quinolone resistance-determining regions (QRDR) were compared to those described for the quinolone sensitive *L. garvieae* strain KL99110 [38].

### Nucleotide Sequence Accession Numbers

The DNA sequences and annotations corresponding to the five plasmids (pGL1 to pGL5) found in *L. garvieae* 21881 have been deposited in the EMBL database under the accession numbers HE650695, HE650696, HE650697, HE651325 and HE651326.

## Results and Discussion

### Plasmid Content and Sequence Analysis

**Figure 1:**
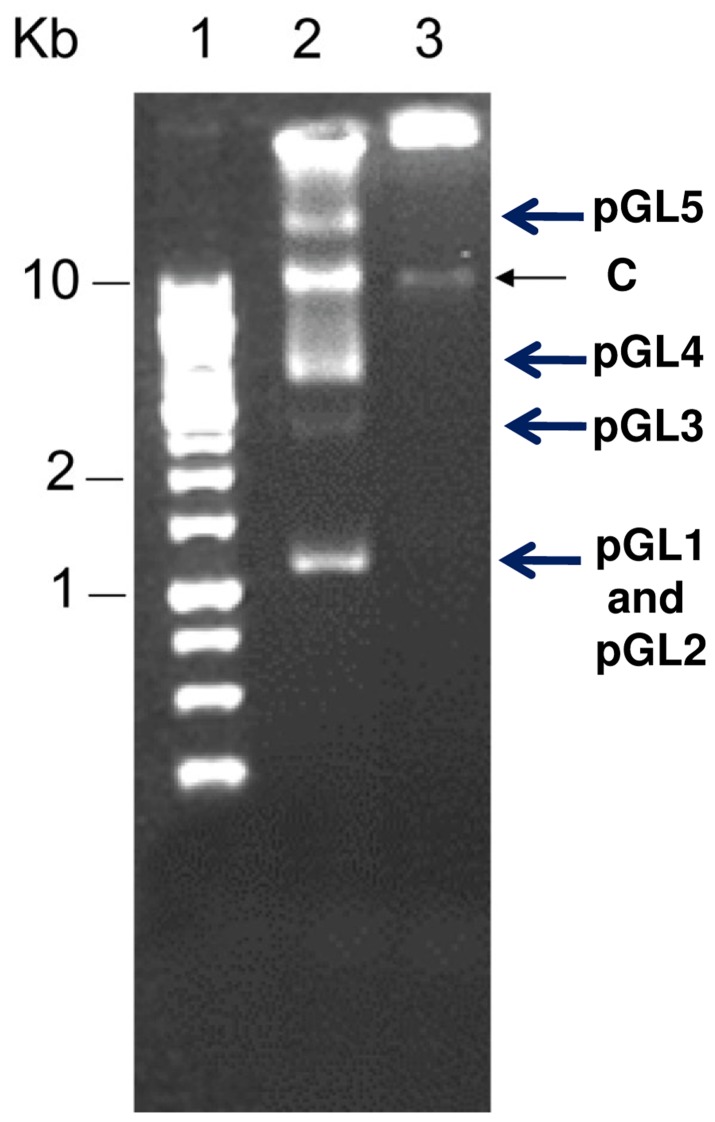
Gel electrophoresis of plasmid DNA from *L. garvieae* 21881 and *L. garvieae* 8831. Line 1, Biotools 1 kb DNA marker, line 2, *L. garvieae* 21881, line 3, *L. garvieae* 8831; C indicates the chromosomal DNA.

The comparison between *L. garvieae* 21881 and the fish isolate *L.* *garvieae* 8831, used as reference, showed that *L. garvieae* 21881 contains five plasmids (Figure 1). We designated these plasmids pGL1, pGL2, pGL3, pGL4 and pGL5 in order of size from small to large; the nucleotide sequence of these plasmids determined molecular sizes of 4,536 bp, 4,572 bp, 12,948 bp, 14,006 bp and 68,798****bp, respectively. These plasmids explain the approximately 0.1 Mb difference in the genome of this strain compared with *L. garvieae* 8831 [16], [20], which is lacking the plasmids (Figure 1). Alignments of pGL1-pGL5 and plasmid pKL0018 present in *L. garvieae* strains isolated from yellowtail (accession number AB290882) [28] were performed using the BLAST two sequences tool (www.ncbi.nlm.nih.gov/blast/bl2seq/wblast2.cgi). Significant DNA sequence similarity (66%) was only found between pGL2 and pKL0018 in the region containing the *repB* gene. The similarity of pGL1-pGL5 plasmids was also searched against the whole-genome shotgun contigs and nucleotide collection databases using the BLASTn program. Overall, the search results showed that the DNA sequences of pGL1-pGL5 plasmids were not present in any of the *L. garvieae* strains for which whole genome sequences are available. These results indicate that pGL1-pGL5 plasmids were solely present in our human strain 21881.

**Figure 2:**
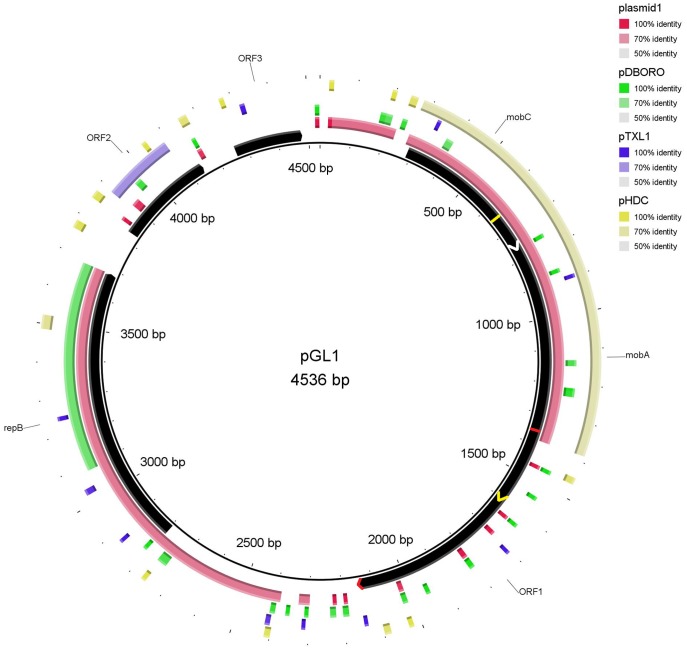
Plasmid map of pGL1 and DNA sequence similarity with other lactic bacteria plasmids. *Lactococcus lactis* subsp. *cremoris* SK11 plasmid1, *Lactococcus lactis* subsp. *lactis* bv. *diacetylactis* plasmid pDBORO, *Leuconostoc mesenteroides* subsp. *mesenteroides* Y110 plasmid pTXL1 and *Tetragenococcus halophilus* plasmid pHDC were those exhibiting the highest coverage/similarity to pGL1. The Orfs are indicated by arrows showing the direction of transcription. The gene annotations are positioned at the midpoint of each gene. Overlapped genes *mobC, mobA* and *orf1* are indicated in white, yellow and red respectively.

**Figure 3:**
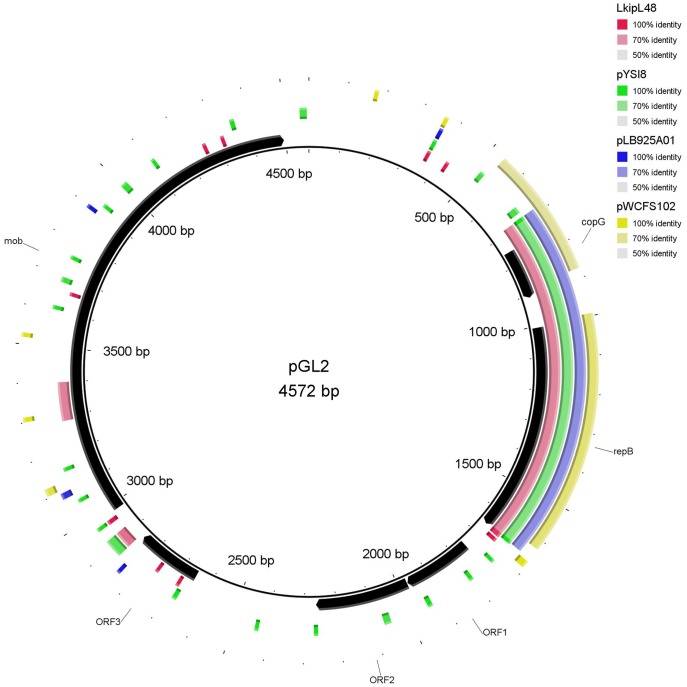
Plasmid map of pGL2 and DNA sequence similarity with other lactic bacteria plasmids. *Leuconostoc kimchii* IMSNU 11154 plasmid LkipL48, *Lactobacillus sakei* plasmid pYSI8, *Lactobacillus brevis* plasmid pLB925A01 and *Lactobacillus plantarum* WCFS1 plasmid pWCFS102 were those exhibiting the highest coverage/similarity to pGL2. The Orfs are indicated by arrows showing the direction of transcription. The gene annotations are positioned at the midpoint of each gene.

**Figure 4:**
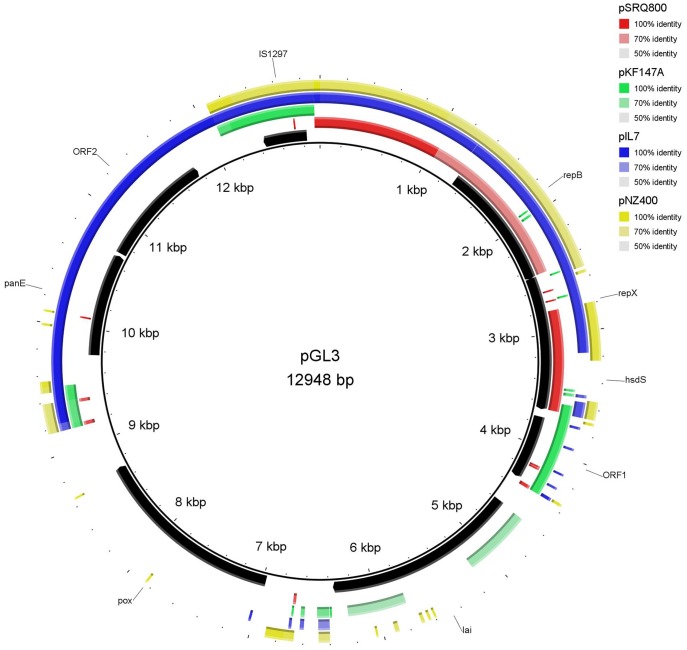
Plasmid map of pGL3 and DNA sequence similarity with other lactic bacteria plasmids. *Lactococcus lactis* plasmid pSRQ800, *Lactococcus lactis* subsp. *lactis* KF147 plasmid pKF147A, *Lactococcus lactis* subsp. *lactis* plasmid pIL7 and *Lactococcus lactis* subsp. *cremoris* plasmid pNZ4000 were those exhibiting the highest coverage/similarity to pGL3. The Orfs are indicated by arrows showing the direction of transcription. The gene annotations are positioned at the midpoint of each gene.

**Figure 5:**
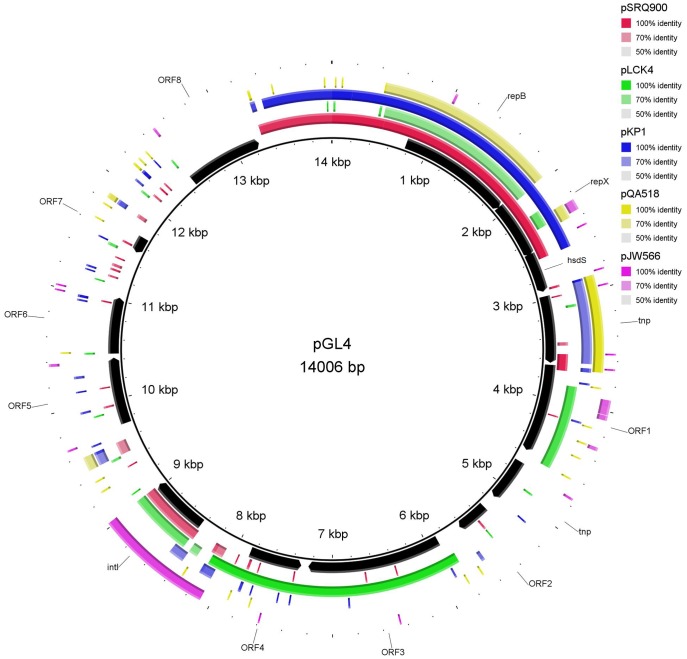
Plasmid map of pGL4 and DNA sequence similarity with other lactic bacteria plasmids. *Lactococcus lactis* plasmid pSRQ900, *Leuconostoc citreum* KM20 plasmid pLCK4, *Lactococcus lactis* subsp. *lactis* plasmid pKP1, *Lactococcus lactis* subsp. *cremoris* A76 plasmid pQA518 and *Lactococcus lactis* plasmid pJW566 were those exhibiting the highest coverage/similarity to pGL4. The Orfs are indicated by arrows showing the direction of transcription. The gene annotations are positioned at the midpoint of each gene.

**Figure 6:**
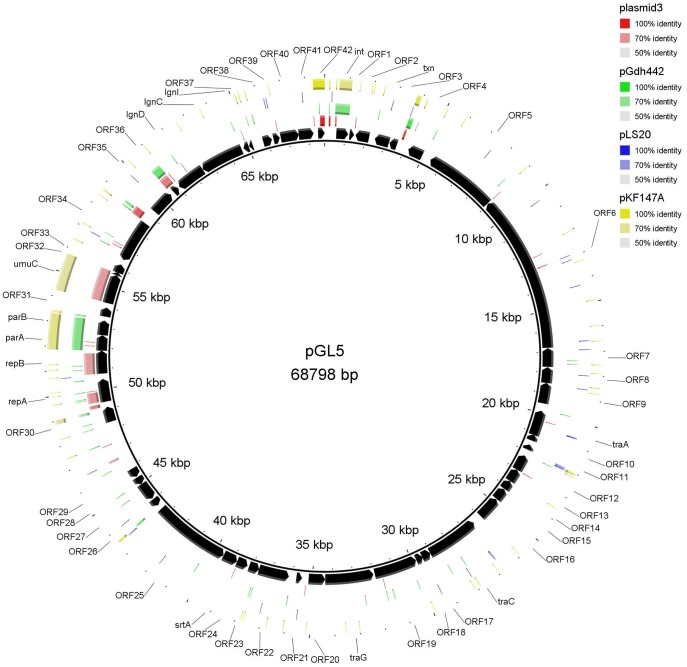
Plasmid map of pGL5 and DNA sequence similarity with other lactic bacteria plasmids. *Lactococcus lactis* subsp. *cremoris* SK11 plasmid 3, *Lactococcus lactis* plasmid pGdh442, *Bacillus subtilis* subsp. *natto* plasmid pLS20 and *Lactococcus lactis* subsp. *lactis* KF147 plasmid pKF147A were those exhibiting the highest coverage/similarity to pGL5. The Orfs are indicated by arrows showing the direction of transcription. The gene annotations are positioned at the midpoint of each gene.

The GC contents of the plasmids were 35.3%, 37.4%, 36.3%, 32.17% and 34.3%, for pGL1 to pGL5, respectively. These values are slightly lower than the values described for *L. garvieae* chromosomal DNA (38–39%) [16]–[20] but are within the range (30–40%) exhibited by most of the lactococcal plasmids [39], [40]. According to the criteria specified above, 91 Orfs were selected and are listed in Tables S1, S2, S3, S4, S5. All of the Orfs had an ATG start codon except for five starting with a TTG and one by a GTG codon. Putative biological functions were assigned to most of the Orfs. The genetic organization of each plasmid is depicted in Figures 2, 3, 4, 5, 6, and the details are presented in Tables S1, S2, S3, S4, S5.

**Figure 7:**
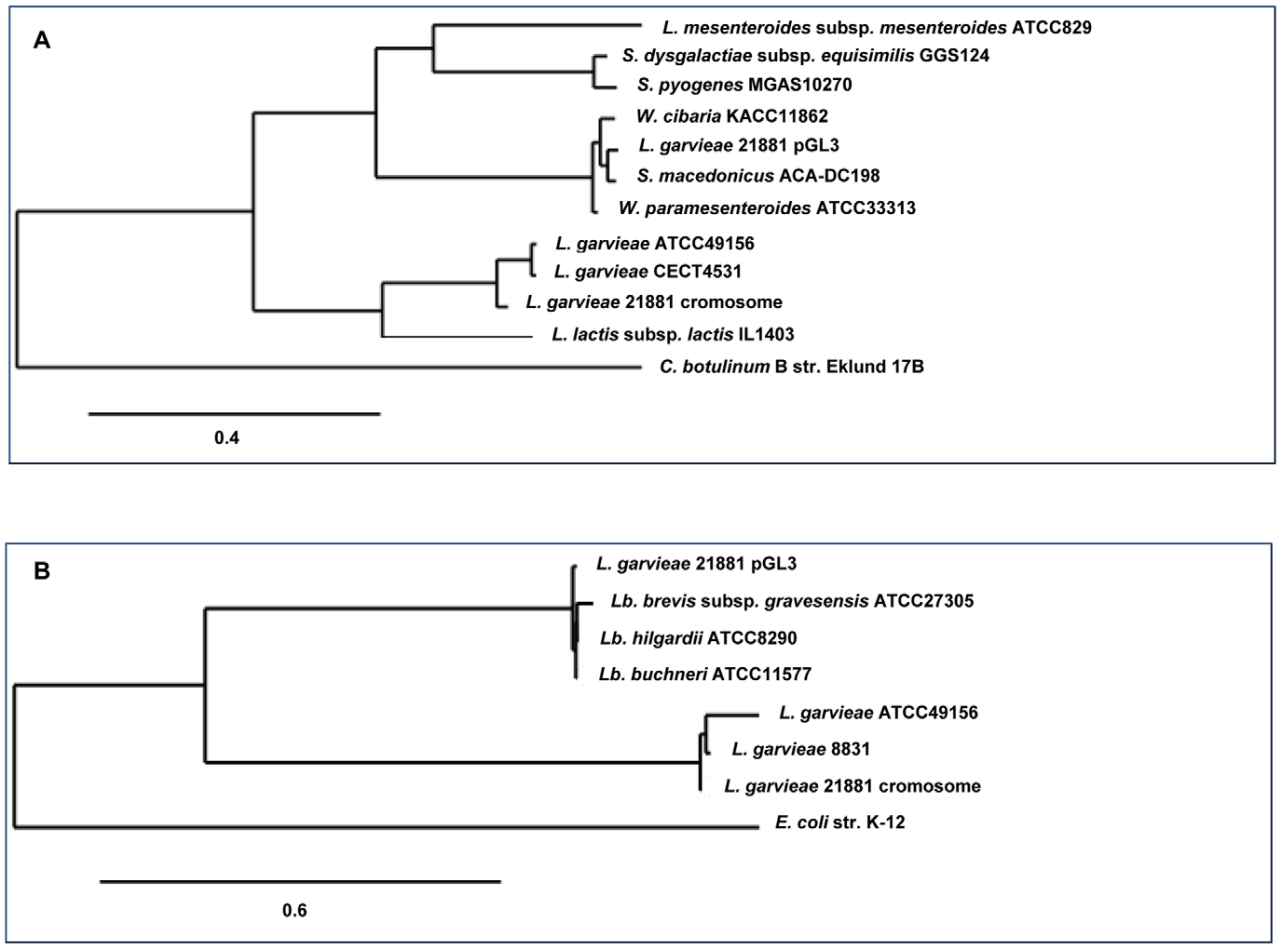
Phylogenetic trees of the *lai* and *pox* genes. The trees are constructed on the basis of the alignment of the DNA sequences of the *lai* (A) and *pox* (B) genes. Genes from *Clostridium botulinum* B str. Eklund 17B and *Escherichia coli* str. K-12 were used as the outgroups.

The majority of the genes present on pGL1-pGL5 exhibited homology with genes located on lactococcal chromosomes (mainly *L. lactis*) or other lactococcal plasmids (Figures 2, 3, 4, 5, 6, Tables S1, S2, S3, S4, S5). However, some genes also shared a high percentage of DNA similarity (>90%) with genes present in other genera of lactic acid bacteria, mainly *Leuconostoc* but also *Lactobacillu*s, *Enterococcus*, *Streptococcus* and *Weissella* (Tables S1, S2, S3, S4, S5). In the case of the pGL4 plasmid, the comparison of the nucleotide sequence of this plasmid with those in the databases revealed two different patterns of similarity (Figure 5). The region located between nucleotides 447 and 2,458 showed 99% DNA sequence similarity to a region of the plasmid pSRQ900 from *L. lactis* [41], coding for RepB and RepX proteins. In contrast, regions located between nucleotides 3,750 and 4,570 and between nucleotides 5,780 and 8,166 showed 99% DNA sequence similarity to DNA regions on the *Leuconostoc citreum* plasmid pLCK4 [42]. Several genes on pGL4 (Table S4) exhibited DNA sequences identities higher than 90% with chromosomal or plasmidic genes of *Streptococcus parauberis* and different *Leuconostoc* species. Two IS elements were identified on the pGL4 plasmid (Table S4); one is similar to a transposase of an *IS946*-like element (an *ISS1*-family element) and the other to an inactive transposase of an *IS30* family element that is commonly found in the genome of *L. citreum* KM20 [42]. Moreover, *orf4* encodes an ATPase involved in DNA repair, which is 99% DNA identical to its homologous gene in *L. citreum* and *orf3* is 100% DNA identical to its homologous gene present in the plasmid pLCK4 (Figure 5) [42]. Likewise, on pGL3, *lai* showed 97% DNA sequence identity to that of *Weissella paramesenteroides* (Table S3) but only 63% DNA identity with the homologous chromosomal gene of *L. garvieae* (accession numbers CBK55550 and AFCC01000000). The genes *pox, orf2* and the sequence of insertion IS129*7* exhibited DNA sequence identity percentages higher than 90% with genes found in *Lactobacillus buchneri, Enterococcus faecalis* and *Leuconostoc* sp., respectively (Table S3). IS elements are known to play an important role in lactococcal gene transfer. In contrast, site-specific recombinases have been studied less in *Lactococcus* and related bacteria. The *int* gene of pGL5 encodes a serine recombinase that is highly identical to the invertase/resolvase subfamily proteins (pfam 002399) from different lactic acid bacteria. This predicted protein shares 77% aa sequence identity to that of *Tetragenococcus halophilus* (Table S5). The presence of mobile elements such as transposon-related genes, IS elements, resolvases, integrases and relaxases, suggests that some of the unique Orfs found within the plasmids may have been acquired from other species via genetic exchange events. The high values of genomic dissimilarity, the differences in CG composition and the presence of transfer mechanisms-associated features for the analyzed genes (Table 1) together with the phylogenetic data (Figure 7), indicate that there have been HGT events between *L. garvieae* and other lactic acid bacteria as has been frequently observed among different species of this group of microorganisms [21], [40], [43]. The fact that most of these lactic acid bacteria (Figures 2, 3, 4, 5, 6, Tables S1, S2, S3, S4, S5) are usually present in dairy products is in line with the hypothesis that some dairy isolates of *L. garvieae* could be responsible of human infections [4].

**Table 1:** Examples of horizontally transferred genes in *L. garvieae* 21881 plamids.

Orf (plasmid)	% GC	Genomic dissimilarity value (δ*) (10^3^)	δ* plot position(1-100%)	HGT mechanism associated feature	Predicted function	Best BLASTP hit
*orf2* (pGL1)	30.67	214.347	94.617	Mobile plasmid	Putative role in replication	*Leuconostoc mesenteroides*
*orf3* (pGL1)	42.92	221.466	89.585	Mobile plasmid	Bacteriocin-like protein	*Enterococcus faecalis*
orf1 and *orf2* (pGL2)	31.84	173.000	93.41	Mobile plasmid	Bacteriocin-like protein andEnterocin A-like protein	*Streptococcus mitis*
*lai* (pGL3)	36.85	104.161	90.80	Transposase	Linoleate isomerase	*Weisella paramesenteroides*
*pox* (pGL3)	42.93	112.894	93.419	Transposase	Pyruvate oxidase	*Lactobacillus buchneri*
*orf4* (pGL4)	26.26	164.409	92.172	Transposase	ATPase involved in DNA repair	*Leuconostoc citreum*
*orf8* (pGL4)	32.95	140.024	90.215	Transposase	Methyl-transferase	*Streptococcus parauberis*
*orf25* (pGL5)	39.49	94.570	96.064	Transposase	Collagen-binding LPXTGprotein	*Enterococcus faecalis*
*orf27* (pGL5)	40.22	163.538	97.523	Transposase	Conserved hypothetical protein	*Enterococcus faecalis*

### Replication and Maintenance Systems

Lactococcal plasmids can replicate by two different mechanisms: theta and rolling circle replication (RCR).

**Figure 8:**
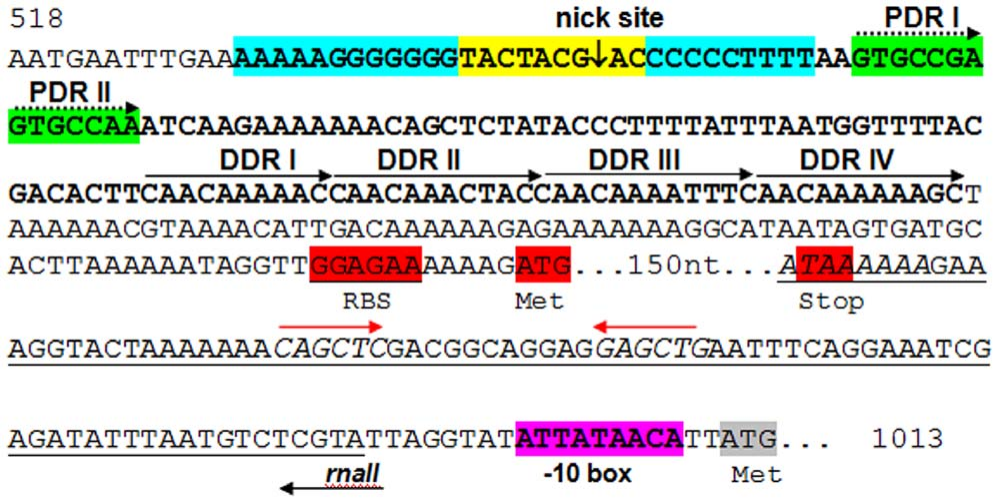
Proposed replication regions (*dso* and *rnaII*) of rolling circle pGL2 plasmid. The *dso* region is indicated in boldface. The *bind* locus contains four tandem distal direct repeats (DDR) which are marked with arrowheads indicating their orientation. The *nic* locus contains: the nick sequence (in yellow) flanked by the inverted repeat structures (in blue) and two proximal direct repeats (PDR) sequences in tandem (in green, with broken arrowheads indicating their orientation). The putative *rnaII* region is underlined. The predicted −10 consensus promoter sequence of *rnaII* is indicated in pink. The predicted transcription terminator region is indicated in italics and the inverted repeat sequences (involved in the RNA II hairpin formation) with red arrowheads indicating their orientation. The presumed ribosome-binding site (RBS) and start and stop codons of the *copG* gene are indicated in red. The start codon of *repB* is indicated in grey.

Plasmid pGL2 appears to be a RCR plasmid, as suggested by the homology of its replication gene to those of other known RCR plasmids, such as pWCFS102 of *Lactobacillus plantarum,* pYSI8 of *Lactobacillus sakei* (Figure 3) [44], [45] and pSSU1 of *Streptococcus suis* [46], all members of the rolling-circle replication pMV158 family. A putative double-stranded origin (*dso*) was identified at coordinates 531–676, which shares 95% identity with the *dso* of the plasmids pSMQ172 of *Streptococcus thermophilus* [47] and pSSU1 of *S. suis* [46]. In the case of the plasmids of the pMV158 family, the *dso* can be physically and functionally separated into two loci, termed *bind* (the binding region of the Rep protein) and *nic* (where the Rep protein cleaves specifically at the nick site, which is conserved among plasmids of the pMV158 family) [48]. Upstream of the *copG* gene, at position 542–550 bp, the conserved 9-mer nick sequence site TACTACGAC was identified. Next to this nick sequence, the inverted repeats IR-I elements that form the hairpin where the 5′-GpA dinucleotide is cleaved by RepB, and the two proximal direct repeats (PDRI and PDRII), were also detected (Figure 8). The locus *bind*, which in the case of pGL2 consists of four tandem 11-bp direct repeats (DR), was located 52 bp downstream of the locus *nic* (Figure 8). Another important element in the replicative process of RCR plasmids is the lagging-strand replication origin (*sso*), which is generally located at a short distance upstream of the *dso*. The *sso*s usually have extensive secondary structure, and unlike the *dso*s, their sequences are generally not homologous among plasmids belonging to the same family [25], [49]. A putative *sso*-like region was found in pGL2 immediately upstream of the *dso*. This region contains several inverted repeats sequences, which could generate stem-loop structures.

**Figure 9:**
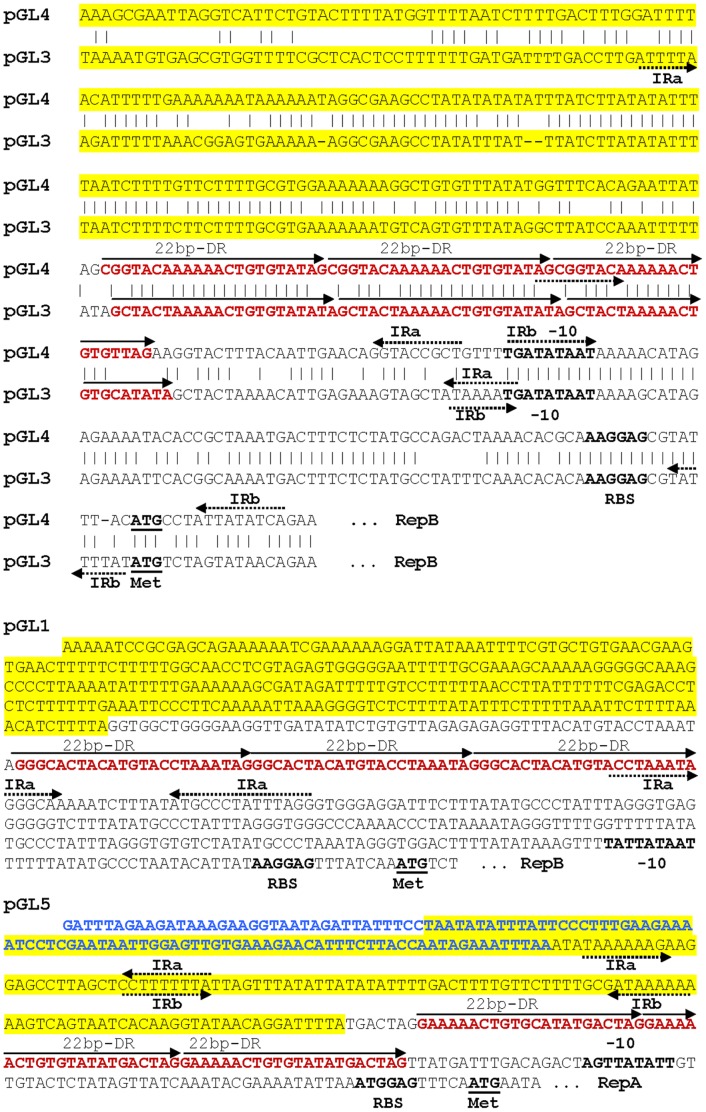
Proposed replication regions of theta-type plasmids pGL1, pGL3, pGL4 and pGL5. Sequence alignment of the upstream region of *repB* genes of only pGL3 and pGL4 showed significant identity (78%). Indicated in yellow are the AT- rich regions. Inverted repeat structures (IR) are represented by opposing broken arrows. The 22-bp directly repeated putative iteron sequences (DR) are indicated in red, and the arrowheads indicate their orientation. Predicted −10 consensus promoter sites and presumed ribosome-binding sites are in boldface. The start codons are underlined. The DNA sequence in blue corresponds to the last nucleotides of *orf30* on pGL5.

The plasmids pGL1, pGL3, pGL4 and pGL5 appear to be theta-replicating plasmids. The replication backbone of these plasmids contains an AT-rich region, the presumed origin of replication, which is located upstream the *rep* gene. This region is followed by three directly repeated sequences of 22 bp (DR, iterons) that are thought to interact directly with the Rep protein to initiate replication. Finally, following the iterons, there are two inverted repeats of 5 to 12 bp, designated as IRa and IRb, that usually overlap the putative −10 and −35 regions of the *rep* gene promoter [41]. The sequence characteristics of this region in plasmids pGL1, pGL3, pGL4 and pGL5 are shown in Figure 9. Plasmid pGL1 carries the *repB* gene (Figure 2) that encodes the replication initiation protein RepB (296 aa). RepB is very similar to the Rep proteins from the *L. lactis* subsp. *cremoris* plasmid 1 (Table S1) and *L. lactis* subsp. *lactis* plasmid pDBORO [22], [50]. Downstream of the *repB* gene (position 3,858) lies *orf2* (Figure 2), which shared 59% aa sequence identity to a hypothetical protein of the plasmid pTXL1 from *Leuconostoc mesenteroides* subsp. *mesenteroides* that has a putative role in replication [51]. The pGL3 and pGL4 plasmids carry a replication gene *repB* that encodes a replication initiation protein RepB of 383 and 386 aa, respectively. Both RepB proteins contain the Rep3 superfamily (pfam01051), and the *L. lactis* RepBC superfamily (pfam06430) conserved motifs and shared a high level of aa sequence identity to initiator proteins of lactococcal theta-replicating plasmids [41], [52]. As often observed in theta-type replicons, a conserved region *rep*X*-hsdS* was observed downstream of *repB* in pGL3 and pGL4. The *repX* gene (also known as *orfX*) is usually overlapped by one or two codons by the *repB* gene [24], [41]. *repX*, very common in theta-replicating plasmids, is not essential for their replication, but in some plasmids, it participates in the control of plasmid copy number, plasmid stability or both [25], [53]. The last gene of this transcription replication-module encodes HsdS, the specific S subunit of a type I restriction modification system [24]. pGL5 carries two replication genes that belong to the *L. lactis* RepBC-terminus superfamily. The *repA* and *repB* genes encode proteins that shared significant similarity (75–80%) with the replication initiator proteins from different lactococcal plasmids such as pSK11L [21], pIL4 [24], pCV56A, [54] and pS7a [55]. Both *rep* genes were found in the same orientation and had a 75% DNA similarity with each other.

Plasmid replication and plasmid stability are closely related processes. Plasmid stabilization requires accurate control mechanisms such as the proper plasmid copy number, plasmid multimer resolution, postsegregational killing and an active partitioning system [25]. In *L. garvieae* 21881, no plasmid loss was observed after approximately 100 generations of culturing in MRS medium without selection pressure, suggesting that the five plasmids possess stabilisation mechanisms. In pGL1, *orf2* encodes a protein with a putative role in replication control similar to that of the pTXL1 plasmid from *L. mesenteroides* [51] (Figure 2, Table S1). pGL2 also contains a plasmid replication copy-number control protein (CopG) similar to that found in *Lactobacillus brevis* (pLB925A01) and other plasmids of the pMV158 family (Figure 3). In these plasmids, two elements, the product of *copG* and a ctRNA (designated RNA II), are involved in the control of the synthesis of the Rep protein [56]. The gene that encodes the ctRNA (*rnaII*) was located between the genes *copG* and *repB* and it reads in the opposite direction. Figure 8 shows the putative *rnaII* gene, with its corresponding putative promoter and terminator regions. Both pGL3 and pGL4 carry the *repX* gene that, in *L. lactis*, participates in the control of plasmid copy number and/or plasmid stability [25], [53]. The genes *parA* and *parB* on pGL5 encode putative partitioning determinants with a high aa identity (76% and 69%, respectively) to homologous proteins ParA and ParB from other *L. lactis* plasmids [24]. This partitioning system is likely to contribute to the segregational stability of pGL5, avoiding the risk of loss during cell division [24], [40].

### Mobilization Genes

Mobility is an essential part of plasmid fitness. Plasmids can be classified into three categories according to mobility: conjugative, mobilizable and nonmobilizable. The protein component common to all transmissible (conjugative or mobilizable) plasmids is the relaxase, a key component in conjugation, because it recognizes the origin of transfer *oriT*. Additionally, conjugative plasmids carry the gene components involved in mating channel formation [57].

pGL1 was predicted to encode two mobilization proteins, MobC (124 aa) and MobA (320 aa), which showed 51% and 67% aa identity to their homologous proteins in the *T. halophilus* plasmid pHDC (Figure 2) [23]. Both the MobA and MobC proteins belong to the group of relaxases (pfam05713) involved in strand separation. A model has been suggested in which MobC acts as a molecular wedge for the relaxosome-induced melting of *oriT* DNA. The effect of MobC on strand separation may be partially complemented by the helical distortion induced by supercoiling. However, MobC extends the melted region through the nick site, thus providing the single-stranded substrate required for cleavage by MobA [58]. The pGL1 plasmid also contains a 234-bp region (bases 65 to 298) with AT-rich content (56.9%) overlapping the promoter sequences of MobC. This region contains the putative origin of transfer *(oriT*) with conservation of the postulated *nic* site (hexamer CTTGCA) just downstream of a conserved pair of inverted repeats (AAAAAAGGCT/TAGCCTTTTTT). The final segment of this region also contains another perfect inverted repeat sequence: TGTTTTTATTTTGTCA/TGACAAAATAAAAACA.

pGL2 was predicted to encode only one mobilization protein, Mob (504 aa), which was 54% aa identical to that of *Lactobacillus acidipiscis* plasmid pLAC1 (YP003650630) and 37% aa identical to the Mob protein encoded by the plasmid pVA380-1 found in *Streptococcus ferus* [59]. This protein is essential for site-specific cointegrative plasmid recombination, and its main biological function may be plasmid mobilization. The alignment of the pGL2 plasmid with *oriT* sequences of other pMV158 family plasmids allowed the identification of a putative *oriT* sequence upstream the *mob* gene. A putative nick site (AGTAAG↓TTA) was found at nucleotides 2903–2911 between the inverted repeat sequences (TAAAGT/ACTTTA) that may form the loop of hairpin [45], [60].

Upstream of the origin of replication (*repB*) of pGL3 and pGL4 (Figures 4 and 5), there is a region of 231 bp that was 98% DNA identical to the transfer origin of the lactococcal plasmids pCD4 [52], pSRQ800, pSRQ900 [41] and pIL7 [24]. These putative *oriT* were located at nucleotides 65–297 on pGL3 and at nucleotides 13,490-13,721 on pGL4 and contain two inverted repeat sequences and a putative nick site CTTGCA. Neither pGL3 nor pGL4 carry any mobilization genes, suggesting that both plasmids would be non-mobilizable.

pGL5 carries three genes encoding proteins homologous to the TraA, TraG/TraD-TrwBVirD4 coupling-protein family (pfam 12696) and TraC-like proteins (also known as VirB4). The *traA* gene encodes a putative relaxase (PRK13878), with a domain that was 34% aa identical and 53% aa similar to a conjugative relaxase of the plasmid pMG2200 of *Enterococcus faecalis*. DNA relaxases are key enzymes in the initiation of conjugative transfer [57]. TraG is thought to be essential for DNA transfer in bacterial conjugation through the mating channel, and TraC is a protein involved in the translocation process [61]. Homologues of TraG and TraC have also been found in staphylococcal (pSK41 and pGO1) and lactococcal (pMRC01) plasmids. Genes encoding TraG and TraC appear to form an operon with other genes (*orf16-orf19*). The protein encoded by *orf16* displayed 42% aa identity to N-acetylmuramoyl-L-alanine amidase of *Streptococcus dysgalactiae* and contains two main enzymatic domains: a glucosaminidase domain (pfam01832) and a cysteine/histidine-dependant amidohydrolase/peptidase (designated CHAP; pfam05257) domain. Hence, Orf16 is predicted to be an exoenzyme able to hydrolyze the cell wall peptidoglycan and could therefore participate in facilitating the passage of DNA across the cell envelope by its peptidoglycan-degradation activity [39]. Similarly, Orf19 shared a 43% aa similarity to the putative membrane spanning protein of conjugative PFR55 plasmid from *Bacillus thurigiensis* [62] and could be involved in mating channel formation. Although no obvious candidate *oriT* region could be found for pGL5, its conjugation region appears to consist of modules that each display sequence similarity to the conjugal transfer determinants of pNP40 and other lactococcal conjugative plasmids [39].

According to these data, pGL1 and pGL2 could be mobilizable plasmids, and pGL5, which may encode the complete set of canonical proteins required for mobilization and transfer, could be a conjugative plasmid.

### Plasmid Defense Mechanisms

Restriction-modification (R/M) systems (types I-IV) are the most common bacteriophage resistance mechanisms found in bacteria. The gene product of *hsdS* in pGL3 and pGL4 was identified as a S-subunit of type I R/M system (Tables S3 and S4), which is responsible for the specificity of endonuclease and methylase activities. The HsdS protein from pGL3 shared 99% aa identity with the homologous protein from pSRQ800 of *L. lactis* subsp. *lactis* (Figure 4), which conferrers resistance against phage P008 by changing the host type I R/M specificity [41]. In a similar fashion, it could be possible that HsdS encoded by pGL4 may be involved in the protection of *L. garvieae* 21881 from bacteriophage attack.

On the other hand, the protein encoded by *orf34* in pGL5 has a unique amino-terminal domain related to the KAP NTPase family proteins (pfam07693). Many of the prokaryotic KAP NTPases are encoded in plasmids. One of their possible functions might be the modification of the bacterial membrane that results in the exclusion of bacteriophages from the plasmid-carrying bacteria [63].

In addition to R/M systems, bacteriocin production is another common defense mechanism against competitive bacteria. The *orf3* and *orf1* genes located on pGL1 and pGL2, respectively, were predicted to encode two putative bacteriocin-like proteins (Table 2). Similarly, the protein products of *lgnD, lgnC, lgnI* and *orf37* on pGL5 appear to also be involved in the bacteriocin production, secretion and immunity (Table S5). These results are in accordance with current studies that confirm the presence of at least one bacteriocin in filtered supernatants from *L. garvieae* 21881 (data not shown).

**Table 2:** Putative virulence factors and defense mechanisms in *L. garvieae* 21881 plasmids.

Plasmid	Orf	Size (aa)	Related protein	Organism	% Identity/Similarity	Accession number
pGL1	*orf3*	72	Putative bacteriocin-like protein	*Streptococcus pyogenes Enterococcus faecalis*	57/66 46/58	ACT32367EFM82101
pGL2	*orf1*	71	Putative bacteriocin-like protein	*Streptococcus mitis*	57/75	CBJ23192
pGL2	*orf2*	97	Bacteriocin- like immunity protein	*L. lactis* subsp. *lactis S. equi*subsp. *equi*	41/63 30/40	YP001032092CAW94771
pGL4	*orf2*	107	Multidrug resistance protein	*Lactobacillus kisonensis Staphylococcus aureus*	76/89 49/76	EHO53128NP863640
pGL5	*txn*	239	Actin-ADP-ribosylating protein. Putative toxin	*Clostridiun botulinum*	24/42	CAA35828
pGL5	*orf5*	119	Mucin-binding LPXTG protein	*Streptococcus anginosus*	34/51	ZP08525680
pGL5	*orf25*	1273	Collagen-binding LPXTG protein	*Enterococcus faecalis* *Enterococcus faecium*	36/51 32/47	ZP05574571 ZP06695453
pGL5	*orf35*	385	Pentapeptide repeats containing protein	Blood disease bacterium	33/49	CCA83758
pGL5	*lgnI*	88	Bacteriocin immunity protein	*Leuconostoc gelidum*	31/57	ZP08478983
pGL5	*orf37*	63	Putative bacteriocin			
pGL5	*orf40*	253	ABC-type multidrug transport protein	*L. lactis* subsp. *lactis*		AEU40217
pGL5	*orf41*	245	ABC-2 membrane transporter	*L. lactis* subsp. *lactis*	99/99	AEU40216

The UmuC-like protein encoded by pGL5 appears to be involved in the replication of damaged DNA (UV protection and mutagenesis) and contains conserved domains corresponding to the Y-family of DNA polymerases, PolY/PolV/umuC subfamily (cd01700); IMS family (pfam00817: UV protection); and DinP (COG0389: nucleotidyltransferase/DNA polymerase involved in DNA repair). The *orf4* gene on pGL4 appears to encode an ATPase involved in DNA repair (Table S4). In addition, pGL3 contains the *lai* gene that encodes a potential linoleate isomerase/hydratase involved in stress tolerance in *Lactobacillus acidophilus* [64] and in the detoxification of linoleic acid effects in *Streptococcus pyogenes* [65]. These functions might help *L. garvieae* cells to adapt to specific environmental conditions.

### Resistance to Drugs and Chemicals

The proteins encoded by *orf3* on pGL4 and *orf42* on pGL5 shared aa identity (100% and 36%, respectively) with efflux proteins associated with resistance to metal ions, such as copper or silver (Tables S4 and S5). The protein of 107 aa encoded by *orf2* on pGL4 showed a Small Multidrug-Resistance domain (pfam 00893) related with proteins involved in the export of a wide range of drugs, toxins and quaternary ammonium compounds [66]. Orf2 shared 49% aa identity (76% aa similarity) with the protein Smr encoded on the plasmid pSK41 from *S. aureus* (accession number AF051917) which is involved in ethidium bromide resistance.

On pGL5, *orf40* and *orf41*, which appear to be in the same operon, apparently encode a two-component drug resistance transport system similar to the ABC-type Multidrug-Resistance transporters. These are a large family of proteins involved in the transport of a wide variety of different compounds, such as sugars, peptides and more complex organic molecules (bacteriocins, antibiotics and chemicals). In bacteria, these transporters usually include an ATP-binding protein and one or two integral membrane proteins [66]. Orf40, a protein of 184 aa (Table 2), exhibited the COG1131 domain belonging to the ATPase component of ABC-type multidrug resistance system (ABC-type MDR); whereas Orf41, with a domain characteristic of the ABC2 membrane superfamily (COG0842; pfam 12698), could be the transporter membrane component. Orf40 and Orf41 shared 66% and 46% aa similarity, respectively, with the transporter system DrrA and DrrB from *Streptomyces peucetius* (accession number M73758) that confers resistance to daunorubicin and doxorubicin [67]. However, it was not possible to determine specifically which substrate would be transported by the Orf40 and Orf41 transporters proteins.

**Figure 10:**
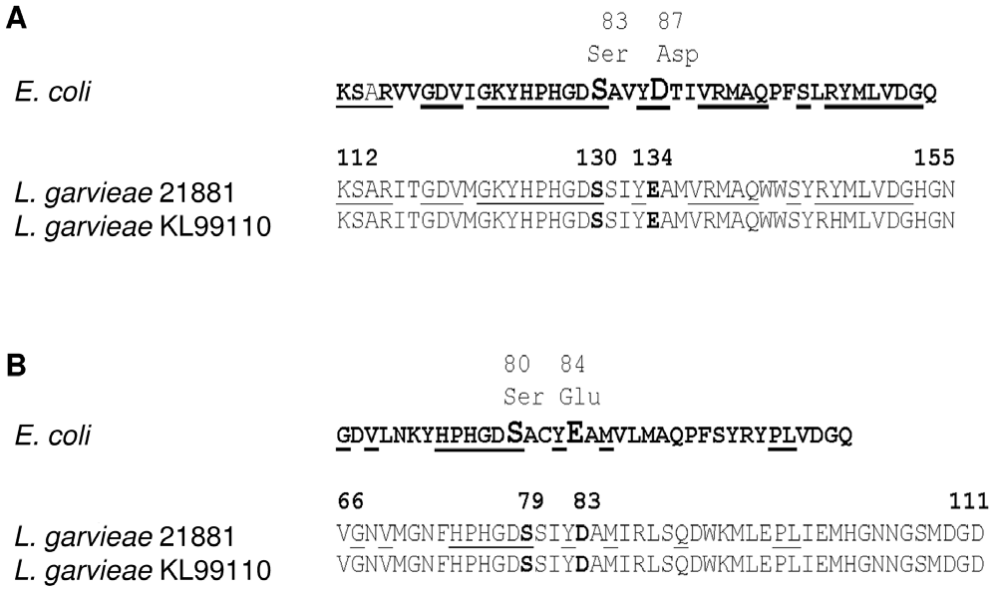
Amino acid sequence of the QRDRs from *L. garvieae* 21881. A) GyrA, B) ParC. Sequences from *L. garvieae* 21881 are compared with those described for the quinolone-sensitive *L. garvieae* strain KL99110. The position of amino acids critical for quinolone resistance (Ser, Asp and Glu) are indicated. Identical amino acids of *L. garvieae* 21881 to the standard QRDR regions for *E. coli* are underlined.

Interestingly, *orf35* on pGL5 encodes a protein of the Pentapeptide Repeat Protein (PRP) family (pfam00805, COG1357). The biological function of most PRP family members is unknown, but these proteins have been increasingly associated with quinolone resistance (Qnr). Qnr is encoded by plasmids in many Gram-negative bacteria and by chromosomal genes in Gram-positive bacteria [68], [69]. *L. garvieae* 21881 was resistant to nalidixic acid (0 mm of diameter of inhibition zone), oxolinic acid (0 mm), flumequine (7 mm), ciprofloxacin (16 mm) and norfloxacin (14 mm) and was sensitive to moxifloxacin (22 mm), in an in vitro disk diffusion susceptibility assay. The main mechanism of quinolone resistance is the accumulation of mutations in the bacterial enzymes DNA gyrase and DNA topoisomerase IV [38], [70]. No mutations were detected in the quinolone-resistance determining regions (QRDRs) of GyrA and ParC of *L. garvieae* 21881 (Figure 10) when compared with the GyrA and ParC sequences of the quinolone susceptible *L. garvieae* strain KL99110 [38]. Mutations in *gyrB* and *parE* are associated with quinolone resistance in other Gram-positive bacteria [70], but were not found in *L. garvieae* 21881 (data not shown). The analysis of the amino acid sequence of Orf35 revealed that critical residues at positions G56, C72, C92, G96, F114, and L159 and loops A and B, which are essential for the interaction with topoisomerases, were highly conserved in Orf35 with respect to the protein sequence of QnrA1, the variant associated with plasmid-mediated quinolone resistance [71]. To our knowledge, this is the first description of a plasmid-born PRP in Gram-positive bacteria, and further studies on the PRP Orf35 are required to elucidate its cellular function.

### Putative Virulence Factors

pGL5 harbors some genes (*txn, orf5* and *orf25*) that encode proteins that could be considered putative virulence factors (Table 2).

The gene *txn* encodes a protein of 239 residues that has the enzymatic domain corresponding to the family of actin-ADP-ribosyltransferases (pfam03496, cd00233). The bacterial ADP-ribosyltransferase toxins are a family of proteins that kill the target eukaryotic cells through the modification of proteins essential for the host organism, playing a key role in the pathogenesis of a variety of bacterial pathogens [72]. In particular, actin ADP-ribosylating bacterial toxins lead to a complete depolymerization or disaggregation of the actin cytoskeleton destroying the microfilament network that contributes to the cytopathic action of these toxins [72], [73]. Txn of *L. garvieae* exhibits the catalytic Glu-X-Glu sequence (residues 199 to 201 in Txn), the NAD binding sites Ser-Thr-Ser sequence (amino acids 156–158 in Txn) and a conserved Arg residue (residing at position 129 in Txn), characteristic of the CT-group of many mono-ADP-ribosyltransferases [73]. Within this group, the toxins could be composed of by a single polypeptide, such as the clostridial C3 exoenzyme and C3-like toxins from *Bacillus cereus* and *S. aureus*, or by two components (binary AB-toxin), such as Iota toxin from *Clostridium perfringens*, the toxin C2 from *Clostridium botulinum,* the *Clostridium spiriforme* toxin, or the vegetative insecticidal protein VIP2 from *B. cereus* [72], [73]. The Txn protein of *L. garvieae* shared 24% aa identity and 42% aa similarity with the C3 exotoxin from *C. botulinum* (accession number CAA35828), indicating that it would belong to the single polypeptide class of C3-family ADP-ribosyltransferases toxins, whose amino acid sequences are considerably diverged [73]. The *orf3* gene, predicted to be located in the same operon as *txn,* encodes a thioredoxin protein, characterized by the TRX fold (amino acids 36–38). Many members of the thioredoxin (TRX)-like superfamily that do not contain the CXXC motif, such as Orf3, function as glutathione peroxidases, GSH transferases, arsenic reductases, transcriptional regulators or chaperones [74]. In contrast to the binary A-B toxins, the cell accessibility of C3-like toxins is unknown in many cases. Therefore, Orf3 might act as a transcriptional regulator of *txn*, as a facilitator of protein folding or in the secretion or cellular uptake of *L. garvieae* Txn toxin across the host membrane. Although ADP-ribosylating toxins have been identified in different species of Gram-positive and Gram-negative bacteria [73], to our knowledge, this is the first time these toxins have been detected in a species of *Lactococcus*.

The genes *orf5* and *orf25* encode two putative surface proteins containing the cell wall-sorting motif LPXTG (Tables 1 and S5) characteristic of Gram-positive cell-wall-anchored surface proteins. The gene *orf5* encodes a protein of 1,179 residues that contains on the carboxy-end a LPXTG-motif (LPQTG) and three mucin-binding protein domains (pfam06458) corresponding to positions 650–750, 850–950 and 1025–1125 of the protein. Bioinformatic analysis of the *orf5* gene product predicted the most likely cleavage site of the signal sequence peptide to be between positions 30 and 31 (ALA-DE). Most commensal and pathogenic bacteria attach to the intestinal mucosal cells through the interaction of adhesins to the mucosal receptors. Thus, the LPXTG-protein encoded by *orf5* could be involved in the binding of *L. garvieae* to mucus from the intestine facilitating further interaction with intestinal epithelial cells. This involvement is especially interesting considering that underlying gastro-intestinal disorders appear to be a factor that contributes to *L. garvieae* infection [14].

The gene *orf25* encodes another putative LPXTG surface protein of 1,271 aa that contains two conserved protein domains: a region on the N-terminal end (KSGKRW), characteristic of signal peptide of some serine-rich and heavily glycosylated proteins, and a collagen-binding protein domain (Cna protein B-type domain; pfam05738), which could facilitate the bacterial adherence to collagen. The Cna protein is an adhesin that plays an important role in the virulence of *S. aureus* [75], [76]. Bioinformatic analysis of this protein predicted the most likely cleavage site of the signal sequence peptide, between positions 42 and 43: ALA-GG. Ten nucleotides upstream of *orf25* is the gene *srtA*, encoding a sortase A (Table S5), which appears to form part of the same operon. Sortase A covalently immobilizes the surface protein to the cell-wall peptidoglycan facilitating the bacterial adherence to the host cell [77]. Thus, the Orf25 protein might provide an advantage to *L. garvieae* for binding collagen substrates and collagenous tissues, such as the collagen-rich heart valves. It is interesting to note that endocarditis is the most common clinical manifestation of *L. garvieae* human infections [1].

LPXTG surface proteins are implicated in the pathogenesis of a number of bacteria, e.g., *S. aureus, Listeria monocytogenes*, enterococci or streptococci playing a functional role in the adherence to host cells [78]–[80]. Thus, the LPXTG surface proteins Orf5 and Orf25 could be implicated in the first interaction step of *L. garvieae* 21881 with human cells facilitating further infection. Genes *orf5* and *orf25* were not detected in any of the *L. garvieae* strains with whole genome sequences available [16]–[19]. *L. garvieae* 21881 also carries other predicted LPXTG-proteins in its chromosome [20]. One of these chromosomal LPXTG-proteins (located between contigs 25–26 from whole sequence of accession number AFCC01000000) showed high aa identity (98%) with the mucin-binding protein (accession number HM852546) present in another *L. garvieae* human clinical strain [27] but absent in fish strains [16], [18], [19], [26]. These data suggest the existence of specific adhesins in human *L. garvieae* isolates and therefore the possibility of different strategies in *L. garvieae* for interacting with host cells. Further studies including more clinical strains will be necessary to further corroborate these results and elucidate the role of these virulence genes in the pathogenesis of *L. garvieae*.

This is the first report on the characterization of plasmids in a human clinical strain of *L. garvieae*. The similarity observed between the *orfs* found in the plasmid genome of this strain and the chromosomal or plasmidic genes of other lactic acid bacteria suggests the existence of horizontal gene transfer events among *L. garvieae* and these bacteria. These plasmids harbor genes related to drug resistance, bacteriocin production and genes that might help *L. garvieae* cells to adapt to specific environmental conditions. Most interesting was the detection of putative virulence genes, such as the gene *txn* that encodes a protein corresponding to the family actin-ADP-ribosyltransferases toxins and the genes *orf5* and *orf25* that encode surface LPXTG proteins with mucin and collagen-binding domains that may be implicated in the adherence to host cells. These results could be useful to understand the factors involved in host invasion and infection of *L. garvieae*.

## Supporting Information

Table S1 **Putative genes identified on pGL1.** Mob proteins were classified into a relaxase (MOB) family according to Smillie et al. 2010.
(DOC)

Table S2 **Putative genes identified on pGL2.** Mob proteins were classified into a relaxase (MOB) family according to Smillie et al. 2010.
(DOC)

Table S3 **Putative genes identified on pGL3.**
(DOC)

Table S4 **Putative genes identified on pGL4.**
(DOC)

Table S5 **Putative genes identified on pGL5.** Mob proteins were classified into a relaxase (MOB) family according to Smillie et al. 2010.
(DOC)
