# Supplementary material for: Characterization of Plasmids in a Human Clinical Strain of Lactococcus garvieae
Source: PLoS One. 2012 Jun 29;7(6):e40119. doi: 10.1371/journal.pone.0040119 (PMC3387028; doi:10.1371/journal.pone.0040119)
Supplement: Table S1 — Putative genes identified on pGL1. Mob proteins were classified into a relaxase (MOB) family according to Smillie et al. 2010. (DOC) [file pone.0040119.s001.doc]

**Table S1.** Putative genes identified on pGL1.

| **ORF** | **Position** (nt) | **% GC** | **Size** (aa) | **Related protein** | **Organism/ Plasmid** | **% Identity*** (aa overlap) |
| --- | --- | --- | --- | --- | --- | --- |
| *mobC* | 287- 661 | 41.86 | 124 | Mobilization protein C | *Tetragenococcus halophilus/* pHDC | 51 (60) |
| *mobA* | 643-1605 | 42.88 | 320 | Mobilization protein A (relaxase) | *Tetragenococcus halophilus*/ pHDC | 67 (167) |
| *orf1* | 1367-2146 | 40.64 | 259 | Putative role in inhibition of recombination | No hits | - |
| *repB* | 2797- 3687 | 34 | 296 | Replication initiator protein | *L. lactis* subsp. *cremoris/* plasmid1 | 78 (232) |
| *orf2* | 3833- 4144 | 32.69 | 103 | Putative role in replication | *Leuconostoc mesenteroides/* pTXL1 | 63 (58) |
| *orf3* | 4261- 4479 | 42.92 | 72 | Bacteriocin-like protein | *Enterococcus faecalis* | 46 (32) |

* Identity lower than 30% has not been considered
