# Supplementary material for: Characterization of Plasmids in a Human Clinical Strain of Lactococcus garvieae
Source: PLoS One. 2012 Jun 29;7(6):e40119. doi: 10.1371/journal.pone.0040119 (PMC3387028; doi:10.1371/journal.pone.0040119)
Supplement: Table S2 — Putative genes identified on pGL2. Mob proteins were classified into a relaxase (MOB) family according to Smillie et al. 2010. (DOC) [file pone.0040119.s002.doc]

**Table S2.** Putative genes identified on pGL2.

| **ORF** | **Position** (nt) | **% GC** | **Size** (aa) | **Related protein** | **Organism/ Plasmid** | **% Identity*** (aa overlap) |
| --- | --- | --- | --- | --- | --- | --- |
| *copG* | 752-907 | 28.84 | 51 | Copy-number control protein | *Lactobacillus brevis*/ pLB925A01 | 66 (33) |
| *repB* | 1005-1658 | 32.92 | 217 | Replication protein | *Lactobacillus plantarum*/ pWCFS102 | 84 (183) |
| *orf1* | 1753-1968 | 34.72 | 71 | Bacteriocin-like protein | *Streptococcus mitis* | 57(16) |
| *orf2* | 1971-2264 | 29.93 | 97 | Enterocin A-like immunity protein | *Streptococcus mitis* | 37 (31) |
| *orf3* | 2654-2857 | 36.76 | 69 | Hypothetical protein | No hits | - |
| *mob* | 2980-4494 | 44.55 | 504 | Mobilization protein | *Streptococcus ferus/* pVA380-1 | 37 (138) |

* Identity lower than 30% has not been considered
