# Supplementary material for: Characterization of Plasmids in a Human Clinical Strain of Lactococcus garvieae
Source: PLoS One. 2012 Jun 29;7(6):e40119. doi: 10.1371/journal.pone.0040119 (PMC3387028; doi:10.1371/journal.pone.0040119)
Supplement: Table S3 — Putative genes identified on pGL3. (DOC) [file pone.0040119.s003.doc]

**Table S3.** Putative genes identified on pGL3.

| **ORF** | **Position** (nt) | **% GC** | **Size** (aa) | **Related protein** | **Organism/ Plamid** | **% Identity*** (aa overlap) |
| --- | --- | --- | --- | --- | --- | --- |
| *repB* | 1321-2472 | 35.5 | 383 | Replication initiator protein | *L. lactis* subsp. *lactis*/ pS7a | 99 (381) |
| *repX* | 2469-3092 | 30 | 207 | Replication associated protein | *L. lactis* subsp. *Lactis*/ pS7a | 98 (203) |
| *hsdS* | 3068-3676 | 36.4 | 202 | Methylase S | *L. lactis*/ pSRQ800 | 99 (200) |
| *orf1* | 3745-4326 | 28 | 193 | Hypothetical protein | *L. lactis* subsp. *lactis*/ pKF147A | 89 (171) |
| *lai* | 4594-6357 | 36.8 | 587 | Putative linoleate isomerase | *Weisella paramesenteroides* | 97 (571) |
| *pox* | 6974- 8713 | 42.9 | 579 | Pyruvate oxidase | *Lactobacillus buchneri* | 99 (574) |
| *panE* | 9768-10706 | 40.4 | 312 | D-2-Hydroxyacid dehydrogenase | *L. lactis* subsp. *lactis* | 100 |
| *orf 2* | 10732-11769 | 41.8 | 345 | Phosphotransferase system, EIIC component family-protein | *Enterococcus faecalis* | 99 (344) |
| IS1297 | 12827-12429 | 38 | 132 | Truncated transposase | *Leuconostoc* sp. | 92 (120) |

* Identity lower than 30% has not been considered
