# Supplementary material for: Characterization of Plasmids in a Human Clinical Strain of Lactococcus garvieae
Source: PLoS One. 2012 Jun 29;7(6):e40119. doi: 10.1371/journal.pone.0040119 (PMC3387028; doi:10.1371/journal.pone.0040119)
Supplement: Table S4 — Putative genes identified on pGL4. (DOC) [file pone.0040119.s004.doc]

**Table S4.** Putative genes identified on pGL4.

| **ORF** | **Position** (nt) | **% GC** | **Size** (aa) | **Related protein** | **Organism/ Plasmid** | **% Identity*** (aa overlap) |
| --- | --- | --- | --- | --- | --- | --- |
| *repB* | 774-1934 | 36.14 | 386 | Replication initiator protein | *L. lactis*/ pSRQ900 | 99 (384) |
| *repX* | 1931-2518 | 34.52 | 195 | Replication associated protein | *L. lactis/* pSRQ900 | 97 (190) |
| *hsdS* | 2506-2910 | 37 | 134 | Methylase S | *L. lactis* susp. *lactis*/ pDR1 | 47 (48) |
| *tnp* | 2961-3641 | 37.30 | 237 | IS946-like transposase | *L. lactis*/ pMRC01 | 100 |
| *orf1* | 3663- 4568 | 32.33 | 301 | Replication initiator protein (repA) | *Leuconostoc citreum*/ pLCK4 | 81 (200) |
| *tnp* | 4703-5149 | 37.55 | 148 | IS30-like incomplete transposase | *Leuconostoc paramesenteroides* | 93 (130) |
| *orf2* | 5295-5618 | 34.87 | 107 | Multidrug resistence protein | *Lactobacillus brevis* | 67 (72) |
| *orf3* | 5882-7246 | 36.7 | 454 | Cation (copper) transporting ATPase | *Leuconostoc citreum*/ pLCK4 | 100 |
| *orf4* | 7856-7319 | 26.2 | 178 | ATPase involved in DNA repair | *Leuconostoc citreum*/ pLCK4 | 94 (167) |
| *intl* | 8436-9026 | 36.88 | 196 | Integrase (tyrosine recombinase) | *L. lactis*/ pSRQ800 | 91 (179) |
| *orf5* | 9740-10417 | 25.51 | 225 | Hypothetical protein | No hits | - |
| *orf6* | 10460-11032 | 22.68 | 190 | Hypothetical protein | No hits | - |
| *orf7* | 11691-11527 | 36.36 | 54 | Hypothetical protein | *L. lactis/* pPF107-3 | 88 (30) |
| *orf8* | 12468-13244 | 32.94 | 258 | SAM dependent methyltransferase | *Streptococcus parauberis* | 96 (247) |

***** Identity lower than 30% has not been considered
