# Supplementary material for: Characterization of Plasmids in a Human Clinical Strain of Lactococcus garvieae
Source: PLoS One. 2012 Jun 29;7(6):e40119. doi: 10.1371/journal.pone.0040119 (PMC3387028; doi:10.1371/journal.pone.0040119)
Supplement: Table S5 — Putative genes identified on pGL5. Mob proteins were classified into a relaxase (MOB) family according to Smillie et al. 2010. (DOC) [file pone.0040119.s005.doc]

**Table S5.** Putative genes identified on pGL5.

| **ORF** | **Position** (nt) | **% GC** | **Size** (aa) | **Related protein** | **Organism/ Plasmid** | **% Identity*** (aa overlap) |
| --- | --- | --- | --- | --- | --- | --- |
| *int* | 569-1147 | 33.3 | 192 | Invertase/Resolvase | *Tetragenococcus halophilus*/ pHDC | 77 (141) |
| *orf1* | 1218-1442 | 33.3 | 74 | Hypothetical protein | No hits | - |
| *orf2* | 2186-1512 | 28.6 | 224 | Hypothetical protein | No hits | - |
| *txn* | 3194-2475 | 31.6 | 239 | Actin-ADP-ribosylating protein. Putative toxin | No hits | - |
| *orf3* | 3596-3222 | 34.9 | 124 | Thioredoxin-like protein | *L. garvieae* | 46 (56) |
| *orf4* | 4944-4207 | 33.3 | 245 | Hypothetical protein | No hits | - |
| *orf5* | 8943-5404 | 38.2 | 1179 | Mucin-binding LPXTG protein | *Streptococcus anginosus* | 34 (185) |
| *orf6* | 16779-8962 | 38 | 2605 | DNA methylase /Helicase (Tn5253 SNF2-related) | *S. pneumoniae* | 39 (709) |
| *orf7* | 17715-16798 | 32.1 | 305 | Hypothetical protein | No hits | - |
| *orf8* | 18510-17731 | 35.3 | 259 | Deoxyribonuclease | *Streptococcus salivarius* | 48 (120) |
| *orf9* | 19523-18510 | 38.2 | 337 | LtrC like-protein (Putative DNA Primase) | *S. epidermidis* | 32 (102) |
| *traA* | 21144-19897 | 38.3 | 415 | Putative conjugative relaxase | *E. faecalis/ pMG2200* | 34 (126) |
| *orf10* | 21431-21126 | 35.3 | 101 | Hypothetical protein | No hits | - |
| *orf11* | 21919-21653 | 32.33 | 88 | Hypothetical protein | No hits | - |
| *orf12* | 23032-22244 | 30.2 | 262 | Hypothetical protein | No hits | - |
| *orf13* | 23686-23033 | 33.2 | 217 | Hypothetical protein | No hits | - |
| *orf14* | 24157-23690 | 36.7 | 155 | Single-stranded DNA-binding protein | *Lactobacillus fermentum* | 38 (51) |
| *orf15* | 24785-24171 | 33 | 204 | Hypothetical protein | No hits | - |
| *orf16* | 25933-24809 | 43.2 | 374 | Immunogenic CHAP protein | *Streptococcus dysgalactiae* | 42 (160) |
| *traC* | 29012-26409 | 36.6 | 867 | Putative translocation protein | *Bacillus subtilis/* pLS20 | 34 (200) |
| *orf17* | 29449-29015 | 33.8 | 144 | Hypothetical protein | *Clostridium beijerinckii* | 33 (40) |
| *orf18* | 29774-29436 | 36.87 | 112 | Hypothetical protein | No hits | - |
| *orf19* | 31927-29795 | 38 | 710 | Hypothetical Daxx protein | No hits | - |
| *traG* | 34373-31995 | 37.11 | 792 | Conjugative protein | *Bacillus subtilis/* pLS20 | 31 (195) |
| *orf20* | 35189-34377 | 37.4 | 270 | Hypothetical protein | No hits | - |
| *orf21* | 35794-35516 | 36.9 | 92 | Zn-peptidase-like family protein | No hits | - |
| *orf22* | 37672-36167 | 33.6 | 501 | Hypothetical protein | No hits | - |
| *orf23* | 38181-37669 | 31.38 | 170 | Hypothetical protein | No hits | - |
| *orf24* | 38817-38272 | 40 | 181 | Hypothetical protein | No hits | - |
| *srtA* | 39546-38827 | 35.5 | 239 | Sortase A | *Streptococcus dysgalactiae* | 50 (107) |
| *orf25* | 43378-39557 | 40 | 1273 | Collagen-binding LPXTG protein | *Enterococcus faecalis* | 36 (134) |
| *orf26* | 43994-43608 | 30.75 | 128 | Hypothetical protein | No hits | - |
| *orf27* | 44931-44050 | 40.8 | 293 | Hypothetical protein | *Enterococcus faecalis* | 36 (54) |
| *orf28* | 45321-44935 | 32.8 | 128 | Hypothetical protein | No hits | - |
| *orf29* | 45819-45364 | 39.73 | 151 | Putative transposase | *Pyrococcus furiosus* | 31 (33) |
| *orf30* | 48355-49104 | 29.6 | 249 | Transcriptional regulator (SOS) | *L. lactis* susp. *lactis* | 51 (104) |
| *repA* | 49365-50489 | 30.3 | 374 | Replication initiator protein | *L. lactis* subsp. *lactis*/ pIL4 | 63 (232) |
| *repB* | 50753-51877 | 27.2 | 374 | Replication initiator protein | *L. lactis* subsp. *lactis*/ pS7a | 64 (234) |
| *parA* | 51874-52635 | 28.5 | 253 | Plasmid segregation | *L. lactis* subsp. *lactis*/ pVF50 | 76 (191) |
| *parB* | 52638-53360 | 28.5 | 240 | Plasmid partition protein | *L. lactis* subsp. *lactis*/ pGdh442 | 69 (165) |
| *orf31* | 53557-54006 | 32.22 | 149 | Hypothetical protein | No hits | - |
| *umuC* | 54228-55664 | 40.2 | 478 | DNA polymerase involved in UV protection | *L. lactis* subsp. *lactis*/ pIL7 | 69 (324) |
| *orf32* | 55661-55828 | 42.26 | 55 | Hypothetical protein | *L. garvieae* unknown protein | 81(39) |
| *orf33* | 55803-56234 | 37.5 | 143 | Thioredoxin-like protein | *L. lactis* subsp. cremoris | 40 (53) |
| *orf34* | 58577-56487 | 27.4 | 696 | Putative KAP family NTPase | *Streptococcus criceti* | 32 (143) |
| *orf35* | 59318-60475 | 29.8 | 385 | Pentapeptide repeats containing protein | Blood disease bacterium | 33 (59) |
| *orf36* | 60661-60957 | 35.7 | 98 | DNA ligase (contains BRCT domain) | *L. lactis* subsp. *lactis*/ pIL4 | 81 (79) |
| *lgnD* | 62459-61032 | 32.42 | 475 | Bacteriocin ABC transporter | *L. lactis* subsp. *lactis* | 57 (272) |
| *lgnC* | 64616-62469 | 34.3 | 715 | Bacteriocin processing and secretion protein | *L. lactis* subsp. *lactis* | 74 (527) |
| *lgnI* | 64988-64722 | 30 | 88 | Bacteriocin immunity | *Leuconostoc gelidum* | 31 (24) |
| *orf37* | 65199-65008 | 38.54 | 63 | Putative bacteriocin | No hits | - |
| *orf38* | 65744-66184 | 26.7 | 146 | LytTr DNA-binding-domain containing response regulator | *L. lactis* subsp. *lactis* | 32 (48) |
| *orf39* | 66265-66579 | 22.22 | 104 | Hypothetical protein | *L. lactis* subsp*. cremoris* | 97 (36) |
| *orf40* | 66625-67527 | 28.87 | 300 | ABC transporter-like protein | *L. lactis* subsp. *cremoris* | 98 (294) |
| *orf41* | 67520-68257 | 26 | 245 | ABC-2 membrane transporter | *Lactobacillus ultunensis* | 51 (125) |
| *orf42* | 68475-68798 | 21 | 107 | Putative cation efflux system protein | *L. lactis* subsp. *lactis*/ pVF21 | 100 |

* Identity lower than 30% has not been considered
